# Recurvatum of the Knee in Cerebral Palsy: A Review

**DOI:** 10.7759/cureus.14408

**Published:** 2021-04-10

**Authors:** David A Yngve

**Affiliations:** 1 Department of Orthopaedic Surgery and Rehabilitation, University of Texas Medical Branch, Galveston, USA

**Keywords:** cerebral palsy, knee, recurvatum, gait, stance, hamstring surgery, ankle-foot orthosis, knee-ankle-foot orthosis

## Abstract

Recurvatum is defined as hyperextension of the knee in the stance phase of gait. Recurvatum knee is a naturally occurring common gait deviation in those with cerebral palsy, along with crouch knee, jump knee, and stiff knee gaits. Early and late recurvatum occur in the first and second halves of stance. Early recurvatum is associated with dynamic calf contraction that raises the heel and pushes the knee into hyperextension as the forefoot contacts the floor. Late recurvatum occurs after the foot is already flat on the floor. As the body weight comes forward over the foot, the tibia stops its forward motion too early as the ankle comes to its range-of-motion limit. The advancing body then moves forward over a hyperextending knee. Surgical hamstring lengthening can have recurvatum as a side effect. There are several strategies to decrease this risk. Medial hamstring lengthening may be safer than combined medial and lateral lengthening. The concept here is that less lengthening or less aggressive lengthening means less recurvatum risk. However, combined medial and lateral lengthening can be reasonably safe from the risk of causing recurvatum if the knee is showing enough preoperative flexion in stance to warrant the increased surgery. More flexion in stance relates to less risk, while less flexion in stance relates to more risk. Knee flexion in stance can be measured. This is done by measuring knee flexion at initial contact and knee flexion in stance in a gait lab or with stop-action video. If there is minimal knee flexion in stance, hamstring lengthening might not be advisable, even if the hamstrings are tight on popliteal angle testing. There are other factors that contribute to recurvatum risk, such as knee hyperextension on static exam, equinus contracture, and jump knee gait. For treatment of recurvatum, the mainstay is the use of ankle foot orthoses set in dorsiflexion. Surgical equinus correction in those with early stance recurvatum can be effective but it is not likely to be effective in those with late stance recurvatum.

## Introduction and background

Recurvatum, or knee hyperextension in gait, is a common finding in adults post-stroke [1] and in adults following prolonged immobilization, physeal arrest, fracture complications, and soft-tissue laxity [2]. In hemiplegic stroke, there can be an incidence of recurvatum of 19.5% [3]. There are review articles on recurvatum following stroke and on adult recurvatum from non-neurologic causes [2]; however, there are no review articles on recurvatum in cerebral palsy, even though it is common [1,4,5]. A search for references specifically devoted to recurvatum in cerebral palsy found 10 articles reporting on a total of 164 patients. Other articles (e.g., articles on hamstring lengthening) did discuss recurvatum to an extent. The purpose of this study is to provide a needed review of recurvatum in cerebral palsy. In this review, we discuss the definition, pathophysiology, natural history, prevalence, incidence following hamstring surgery, strategies to minimize recurvatum following hamstring surgery, treatment, and future research.

## Review

Definition

Common, naturally occurring common gait abnormalities of the knee in cerebral palsy are crouch knee, jump knee, stiff knee, and recurvatum knee [5]. Recurvatum in cerebral palsy occurring without previous surgery is called primary recurvatum [6]. The number of degrees of knee hyperextension needed to qualify as recurvatum varies in the literature between 0° and 5°. Recurvatum can be divided into moderate recurvatum at 5° to 15° and severe recurvatum at >15° of hyperextension [6].

Pathophysiology

Biomechanics

Recurvatum occurs when the position of the weightbearing line from the center of mass of the body to the center of pressure on the floor lies anterior to the knee-joint axis, creating a hyperextension force [7]. During the gait cycle, the recurvatum starts when the tibia stops moving forward. When this happens, the body weight pivots forward over a relatively stationary knee joint. The advantage of a hyperextended knee is that it creates a stable post which will not collapse and cause a fall [7]. However, recurvatum gait limits stride length and velocity, impairing smooth transfer of momentum and reducing energy efficiency [5]. When the recurvatum occurs in the first half of stance phase, it is called early recurvatum; in the second half of stance phase, it is called late recurvatum [7,8]. The biomechanics and physiology of early and late recurvatum are different.

Early Stance Recurvatum

**Figure 1:**
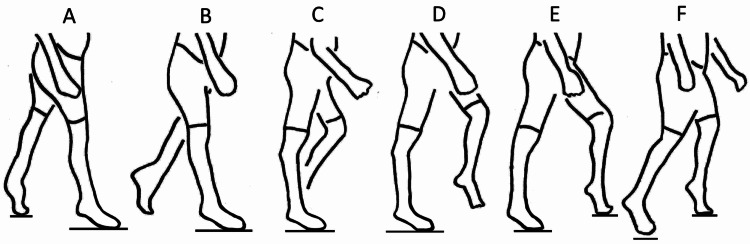
Early stance recurvatum. (A) The gait cycle starts with initial contact of the right foot. Both feet are in equinus. (B) As the body weight transfers to the right leg, the right ankle is still in equinus. The right knee extends sooner than normal in response to the plantar flexion-knee extension couple. (C) The right knee is already in recurvatum by the time mid-stance is reached. (D) As the left leg swings forward, the right knee continues in recurvatum. (E) As the left foot contacts the floor and starts bearing weight, the right knee starts to flex as the hip flexes. (F) The gait cycle ends as the right foot lifts off the floor. Without weightbearing, the recurvatum resolves.

In early stance recurvatum, there is active contraction of the calf muscles, which often lifts the heel as it causes knee extension due to weightbearing through the forefoot (Figure 1). This strong role of excessive plantarflexion characterizes early recurvatum [6, 8-10]. In one study, only 44% of patients with early recurvatum ever obtained heel contact [8]. The strong plantarflexion creates a stronger extensor torque at the knee in those with early recurvatum, compared to those with late recurvatum [1]. In early recurvatum, the soleus has an exaggerated stretch reflex, which causes a dynamic soleus contraction; this has been confirmed by electromyography [1]. The knee can hyperextend forcefully with early recurvatum, as the weight-bearing line swings from posterior to anterior of the knee. Jump knee gait is another gait abnormality that also has dynamic calf contraction. In jump knee gait, the knee is flexed on initial contact, but then extends in mid-stance [5]. Many with jump knee gait will never develop recurvatum; however, some do. In one study, those who had jump knee gait before hamstring release were more likely to develop recurvatum, and it was more likely to be long-lasting. All those with persistent recurvatum at eight-year average follow-up had jump knee gait preoperatively, despite “meticulous and successful equinus correction” [11]. Jump knee gait can also be accompanied by a stiff knee pattern [12]. This is consistent with the finding of increased universal tone with jump knee gait [5]. The overall concept of the leg with early recurvatum is neurologically modulated overactivity favoring both ankle plantarflexion and knee extension [12]. There is an exaggerated response to weightbearing, with a combination of spasticity and extensor tone patterning at the ankle and knee.

Late Stance Recurvatum

**Figure 2:**
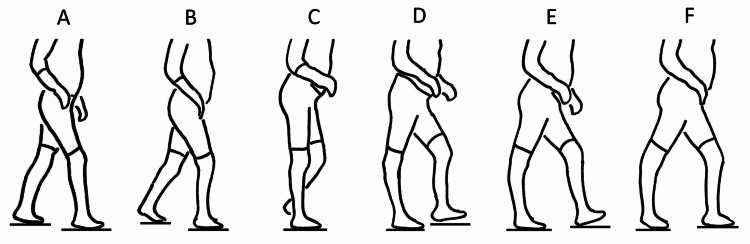
Late stance recurvatum. (A) The gait cycle starts with initial contact of the right foot. The left heel will start to rise only after the right foot has made secure contact and has started to accept weight. (B) Weight continues to shift to the right leg. (C) At mid-stance, the tibia is planted on the floor through a rigid ankle. The tibia is blocked from moving forward. (D) As the body weight continues forward over the fixed tibia, the knee hyperextends. (E) While the left leg swings forward and makes initial contact, the right forefoot and heel remain planted. Normally, the right heel would be rising at this point. (F) The right heel rises only after the left foot has made full contact and started to accept weight.

In late stance recurvatum, the calf muscles are not as active as they are in early stance recurvatum, and the heel will always contact the floor during stance (Figure 2). In stance, as the tibia moves forward, the ankle dorsiflexes until it reaches its range-of-motion limit. At that point, the tibia stops moving forward, but the heel stays down, allowing the recurvatum to start [7,8]. The knee can hyperextend more gently than with early recurvatum, as the increased forces of weight acceptance associated with early stance have passed. In normal gait, the stance foot transitions from foot flat to the forefoot by lifting the heel well before the opposite swing foot contacts the floor. In the late recurvatum group, the stance heel will stay down until after opposite foot-strike occurs and weightbearing has started to shift to the opposite side.

See Appendix for additional commentary on the physiology of early and late stance recurvatum.

Natural history

There is a tendency for some cases of recurvatum to resolve over time. One study reported an incidence of recurvatum at one year following hamstring release surgery of 35%. At three-year follow-up, the incidence was 13%, and at eight-year average follow-up, the incidence was 12% [11]. Another study reported an incidence at one year following hamstring release of 5%. At two-year follow-up in the clinic, the incidence appeared to be 0% [13].

Bony deformity is a potential issue when limb alignment is compromised in the growing child. Concerning recurvatum, a 1972 report stated that proximal tibia growth deformity can occur “when significant hyperextension is ignored” and that an osteotomy may be required, without providing further detail [4]. Knee pain is also an area of potential concern. Knee pain is reported when recurvatum occurs in adult patients with post-trauma etiologies [2]. Knee pain is also reported when recurvatum occurs in adult hemiplegic stroke patients, with an incidence of 11.5% of those with recurvatum [3]. However, the literature in the last 20 years has not contributed any cases of bony deformity or knee pain associated with cerebral palsy and recurvatum, to my knowledge.

Prevalence without previous surgery

It is well established in the literature that recurvatum occurs in children with cerebral palsy who have had no previous lower-extremity surgery. Pooling data from the reports from three gait laboratories, 75 patients have been reported who had recurvatum and cerebral palsy but no previous lower extremity surgery. Average age was 7.8 years (3.9) [6,8,14]. Two of those studies divided patients into those with moderate recurvatum of 5° to 15° and severe recurvatum of >15°. When the data from those two studies were combined, there were 34 patients with moderate and 12 with severe recurvatum [6,8]. One study reported a prevalence of recurvatum without previous lower-extremity surgery of 8% from a database of 463 gait laboratory patients. The prevalence was 5.2% for moderate and 1.5% for severe recurvatum. Of the 30 patients studied, 12 had bilateral recurvatum and 18 had unilateral [6]. The Gross Motor Function Classification System level for these studies was I‑III.

Incidence following hamstring surgery

Recurvatum Incidence Without and With Hamstring Surgery

The top of Table 1 demonstrates a prevalence of 8% of recurvatum incidence with no previous lower-extremity surgery, illustrating the natural occurrence in those with cerebral palsy [6]. At the bottom of the table is remarkable evidence that functioning hamstrings have a protective role against the development of recurvatum. The recurvatum rate following hamstring transfer, as done between 1966 and 1983, was 40% [15]. The remainder of the table deals with recurvatum rates following medial hamstring lengthening and following medial and lateral hamstring lengthening. Medial and lateral hamstring surgery is more surgery than medial only, and therefore might be expected to yield higher recurvatum rates. However, there was only a slight trend for increased recurvatum following medial and lateral lengthening when the data from seven studies and 531 patients are combined. Certain individuals are predisposed to develop recurvatum and will develop it even without prior hamstring surgery. Others have some partial predisposition and might develop recurvatum following hamstring lengthening. Still others are not predisposed to develop recurvatum and may never develop it.

**Table 1:** Recurvatum incidence without and with hamstring surgery.

	Definition	Cases/total	%	Reference
Primary				
No previous surgery	≥ 5 degrees	37/463	8%	Klotz 2014
Following surgery				
Medial hamstrings	> 5 degrees	0/18	0%	Marron 2020
	≥ 5 degrees	2/41	5%	Davids 2019
	> 0	1/16	6%	Kay 2002
	≥ 3 degrees	6/87	7%	Dhawlikar 1992
	Undefined	8/57	14%	Wijesekera 2019
	> 5 degrees	21/60	35%	Dreher 2012
Cumulative total		38/279	14%	
Medial and lateral hamstrings	≥ 3 degrees	4/39	10%	Dhawlikar 1992
	Undefined	22/144	15%	White 2019
	> 0	5/21	24%	Kay 2002
	> 5 degrees	8/30	26%	Marron 2020
	> 5 degrees	4/18	33%	Dreher 2012
Cumulative total		43/252	17%	
Hamstring transfer	Undefined		40%	Campos da Paz 1984

Knee Flexion in Gait

Recurvatum incidence is related to preoperative knee flexion in stance. There are three factors to consider here, degree of knee flexion, dose of hamstring surgery, and resultant recurvatum incidence. A smaller surgery and the presence of a higher degree of knee flexion are protective against developing recurvatum. The gait data from each of the studies were pooled to determine the average preoperative knee flexion at initial contact and at mid-stance, as well as the resultant recurvatum rates (Table 2). The cases with medial hamstring lengthening formed one group, and medial and lateral hamstring lengthening formed a second group.

**Table 2:** Recurvatum incidence related to preoperative knee flexion in gait. SD = standard deviation

Preoperative knee flexion in gait		Cases/total	%	Reference
Initial contact	Mid-stance			
Mean (SD)	Mean (SD)			
Medial hamstrings				
41 (9) degrees	29	0/18	0%	Marron 2020
28 (8)	13 (10)	2/41	5%	Davids 2019
38 (17)	20 (12)	1/16	6%	Kay 2002
34 (12)	30 (13)	8/57	14%	Wijesekera 2019
36 (17)	27 (18)	21/60	35%	Dreher 2012
Cumulative averages/study				
34	24	32/192	17%	
Medial and lateral hamstrings				
Not stated	37	22/144	15%	White 2019
39 (11)	27 (15)	5/21	24%	Kay 2002
42 (11)	26	8/30	26%	Marron 2020
45 (14)	42 (20)	4/18	33%	Dreher 2012
Cumulative averages/study				
42	33	39/213	18%	

The results for the medial hamstring lengthening group showed a mean knee flexion at initial contact of 35° and a mean knee flexion at mid-stance of 24°. There was a resultant recurvatum rate of 17°. The results for the medial and lateral hamstring lengthening group showed more knee flexion, with a mean knee flexion at initial contact of 42°, and a mean knee flexion at mid-stance of 33°. There was a resultant recurvatum rate of 18°. This suggests that surgeons are selecting the more severely flexed knees for the bigger combined surgery, with little change in the resulting recurvatum rate. It indicates that a combined medial and lateral lengthening might be reasonably safe from the standpoint of causing recurvatum if there was enough knee flexion to warrant the additional surgery.

Operative and Postoperative Protocols

The relation of operative and postoperative protocols to the incidence of recurvatum is unclear because of insufficient data, but there was a trend for lower recurvatum rates with less surgery and less postoperative treatment (Table 3). Three papers described recurvatum rates and protocols [11,13,16]. There was a trend for a lower recurvatum incidence of 5% to 10% in the two studies with limited range-of-motion goals in surgery [13,16]. In the third study, there was a higher recurvatum rate with a higher corrective surgical goal, which was a popliteal angle of 20° [11]. In this study, there was also a more corrective postoperative protocol. Night splints were used for six months and, in cases with residual knee-flexion contracture, serial casting was performed. In this study the recurvatum rates were higher at 35% for medial and 33% for medial and lateral lengthening.

**Table 3:** Operative and postoperative protocols related to recurvatum incidence. ROM = range of motion

Operative ROM goal/postoperative plan	Cases/total	%	Reference
Medial hamstrings			
Not forcing/slow stretching	2/41	5%	Davids 2019
Straight-leg raise 70 degrees/cast	6/87	7%	Dhawlikar 1992
20 degrees popliteal/casting to full extension if needed, night splints for 6 months	21/60	35%	Dreher 2012
Medial and lateral hamstrings			
Straight-leg raise 70 degrees/cast	4/39	10%	Dhawlikar 1992
Not stated/knee immobilizers or casts for 4-6 weeks	22/144	15%	White 2019
20 degrees popliteal/casting to full extension if needed, night splints for 6 months	4/18	33%	Dreher 2012

Strategies to minimize recurvatum incidence following hamstring surgery

Operate Only on Those With Significant Knee Flexion in Gait

Evaluation of preoperative knee range of motion in gait is a strategy to minimize recurvatum following hamstring release. Even though many studies did report on static knee range-of-motion measurements such as popliteal angle, it was the knee angle in gait that determined if hamstring lengthening was indicated, not the static measurements. There was one older study that did not include gait data, but in this study, the primary indication was still gait-related: “knee flexion deformity severe enough to impair the patient’s ability to walk” [16]. This is because there are many with knees that are tight to the popliteal angle test, yet they have good knee extension in the stance. This confirms that popliteal angle tightness alone as an indication for hamstring lengthening could lead to overcorrection into recurvatum.

In a retrospective review of 109 patients who had medial and lateral hamstring lengthening, the finding of preoperative knee flexion in stance of 20° or less in males predicted postoperative knee hyperextension (83% accuracy) using receiver operating characteristic curve analysis. The predictive ability did not hold for females [17]. This is consistent with the findings of a 15-member panel, which reported a knee angle of >30° at initial contact and a knee angle of >20° in stance as consensus indications for hamstring lengthening [18]. However, those recommendations are not universal; in two other studies, the stated indications for hamstring lengthening were smaller, with knee flexion at initial contact ≥20° and knee flexion in stance ≥10° [11,19]. Furthermore, a close look at the published studies summarized in Table 2 reveals a spread in preoperative knee angles and standard deviations such that it is doubtful if anyone is using the 30° and 20° consensus knee angles for anything more than a rough guideline. The panel report was published recently, so it could not have been a guide for those studies. However, it brings to light that there have not been widespread range-of-motion standards in use during the last two decades. More studies are needed on this topic, but at a minimum, the literature does currently support that if there is less than 20° of knee flexion at initial contact and less than 10° of knee flexion in stance, hamstring lengthening might not be a good idea because of the risk of recurvatum, even though the hamstrings may be tight on popliteal angle testing. These knee angle determinations can be obtained from a gait lab. Alternatively, knee angles can be measured using a tripod-mounted video camera along with a software program that allows frame-by-frame visualization [20].

Less Aggressive Surgery

There is a trend for doing fewer hamstring lengthening surgeries because of concern for producing increased pelvic tilt [18]. That caution would likely reduce the incidence of recurvatum as well. In addition to not doing hamstring release in certain circumstances, doing hamstring release less aggressively has also been advocated. One study recommended not to use force with hamstring lengthening [13]. Another study focused on limiting straight leg-raising in surgery to 70° [16].

The concept of lengthening the medial hamstrings only has been voiced as a less aggressive option to reduce the risk of recurvatum [21]. There is partial truth to this concept. On one hand, if the goal is to minimize the risk of recurvatum, less lengthening would be better. On the other hand, published articles, when analyzed as a group, do not indicate that combined medial and lateral lengthening has an increased outcome of recurvatum (Table 1). The likely reason, according to the data, is that surgeons tend to reserve medial and lateral surgery for those with more flexion deformity, as determined by preoperative knee flexion at initial contact and in stance. Additional flexion deformity would have a protective effect against recurvatum, allowing the bigger surgery to be more safely tolerated (Table 2).

Treat Equinus

First, consider not correcting equinus. Leaving equinus present while working to correct flexed knee gait could contribute to better correction of the knee flexion through the plantar flexion-knee extension couple. On the other hand, leaving equinus present could lead to recurvatum. Calf spasticity seen after hamstring lengthening is associated with knee hyperextension [22].

Second, consider correcting equinus. Correcting equinus while working to correct flexed knee gait could contribute to less correction of the knee flexion through less of a plantar flexion-knee extension couple. On the other hand, correcting equinus could decrease the chance of developing recurvatum.

What does the literature say? One study suggests that if hamstring lengthening is under consideration, then the calf should be checked for tightness. If the calf is tight, consideration should be given to addressing that issue, to decrease the chance of recurvatum developing [8]. Another study suggests that if medial and lateral hamstrings are to be lengthened, calf spasticity should be evaluated. If present, ankle bracing with or without calf botulinum toxin injections should be considered. This might decrease the chance of recurvatum developing [22].

Quadriceps Tightness

At the knee, it is important to have control of flexion and extension. Flexion control through the hamstrings is important to prevent hyperextension. Quadriceps spasticity is a force that favors hyperextension [4,9,10]. Excessive quadriceps activity is not a major cause of recurvatum in those with cerebral palsy; however, two cases have been reported [4,7]. In a series of 12 children with recurvatum treated by ankle-foot orthoses, 11 had the recurvatum corrected and one did not. That child had electromyographic activity of the quadriceps and the gluteus maximus that was prolonged and persistent through the time that the recurvatum was occurring [7].

It is interesting to explore the concept that rectus femoris surgery at the time of hamstring release might have a protective effect against the development of recurvatum by decreasing knee-extension forces. However, there is no evidence of any protective effect. Looking at published studies, the number of patients who had distal rectus surgery concurrent with hamstring lengthening varies widely. In a review of six hamstring-lengthening studies published between 2012 and 2020, the percentage of hamstring patients having concurrent rectus surgery varied from 0% to 91%, with a study average of 51% [11,17,21,23-25]. There was no obvious effect of the rectus surgery protecting from the occurrence of recurvatum. On the contrary, the paper with the highest percentage of concurrent rectus surgery also had the highest recurvatum rate. They had a 91% rate of concurrent rectus surgery and a 35% rate of recurvatum. However, they also focused on getting more hamstring lengthening during surgery and postoperatively [11].

Knee Hyperextension

The relationship between knee hyperextension on static exam and recurvatum in gait was reported [8]. Looking at gait-laboratory files, 60 patients were identified who had 5° or more of hyperextension on static exam. None had previous lower-extremity surgery. Of those, 15 (25%) walked with knee hyperextension >0° and 45 did not. This incidence is greater than the published baseline incidence of 8% [6]. The implication is that static knee hyperextension is a risk factor for recurvatum in gait in those who have not had lower-extremity surgery. A further implication is that static knee hyperextension is a risk factor for the development of recurvatum following hamstring release. The opposite implication is that a static knee-flexion contracture would be protective against the development of recurvatum following hamstring release, at least for as long as that contracture would persist.

A variation on this theme is illustrated in a study of patients with knee-flexion contracture corrected with various combinations of distal femoral extension osteotomy and patellar tendon advancement. Before corrective surgery, 0% had recurvatum gait and 100% had flexed knee gait. Following surgery, six of 73 (8%) had 5° to 15° of recurvatum by motion analysis [26]. This illustrates that the preoperative flexed knee state protected against recurvatum. When that knee flexion was corrected, recurvatum developed in some.

Treatment of recurvatum

Nonoperative Treatment

Taping has not been shown to be effective in the treatment of recurvatum in patients with cerebral palsy [27]. Also, treatment with botulinum toxin was not effective [28]. Shoe modifications have been recommended for recurvatum associated with ataxic or athetoid cerebral palsy. A heel flair is added to the shoe. This works at heel-strike to provide a force to flex the knee [29]. The use of ankle-foot orthoses has also been described. In a study of 12 children with cerebral palsy and recurvatum, 11 had correction while wearing ankle-foot orthoses set at 7° to 10° of dorsiflexion, as documented in a gait lab [7]. Another study reported that ankle-foot orthoses were used to control knee recurvatum for long-distance walking at an eight-year average follow-up [11]. Also, the use of knee-ankle-foot orthoses has been described. In one study, supracondylar knee-ankle-foot orthoses were used in five patients (mean age, 3.5 years) with cerebral palsy and recurvatum. They all had knee hyperextension and heel rise, suggesting that they had jump gait. The orthotic treatment was reported to be effective [30].

Operative Treatment

In one study, calf lengthening improved recurvatum in some, but not all, patients. In the group with early stance recurvatum, the knee position in stance was improved by 11°. The group with late stance recurvatum did not have significant improvement [31]. There was a study of adult patients without neuromuscular conditions, but with recurvatum caused by fracture malunion, growth-plate problems, and ligamentous laxity. Anterior opening wedge proximal tibial osteotomy was described as a reliable treatment [2]. I have found no reports of this method in cerebral palsy patients.

Future research

Anterior Pelvic

Hamstring lengthening has two long-term risks to balance, anterior pelvic tilt, and recurring knee-flexion contracture. What is the best way to balance these for the best long-term patient outcomes? What is the long-term natural history of increased anterior pelvic tilt? Does it lead to back pain or spine problems? If so, with what incidence and severity?

Hamstring Length

Can modeling of hamstring length and velocity predict recurvatum? With gait analysis and computer modeling, the length and velocity of the hamstrings can be estimated throughout the gait cycle. Could such modeling be a screening modality before proposed hamstring-release surgery? If so, could the hamstring-release plans then be modified or cancelled to prevent the onset of recurvatum following the surgery?

Epiphysiodesis

Anterior distal femoral epiphysiodesis has been used with positive results for the correction of knee-flexion deformity. Static knee flexion to exam and knee flexion in stance can be improved. Does this procedure have a recurvatum risk? Can it replace hamstring release as a more sensible option? In what circumstances?

Semitendinosus Only

There are patients with problematic hamstring tightness, but with minimal crouch at the knee in stance. They do not have the degree of crouch that would indicate a full medial hamstring release. Could a semimembranosus sparing medial hamstring release be an effective low-dose option in these cases, such as release of semitendinosus only, or of semitendinosus and gracilis?

## Conclusions

Recurvatum occurs when the position of the weightbearing line from the center of mass of the body to the center of pressure on the floor lies anterior to the knee axis, creating a hyperextension force. When the recurvatum occurs in the first half of the stance phase, it is called early stance recurvatum; in the second half of the stance phase it is called late stance recurvatum. When recurvatum occurs in those without previous surgery, there are several factors to look for: knee hyperextension to static exam, spasticity and extensor tone in the calf muscles, and limitation of ankle dorsiflexion range of motion. There is a tendency for some cases of recurvatum to resolve over time. Those with long-term recurvatum often have jump knee gait refractory to surgical treatment and use ankle-foot orthoses for walking long distances. Functioning hamstrings have a protective role against the development of recurvatum. Recurvatum can occur after surgical hamstring lengthening. More lengthening results in a higher rate of recurvatum. Evaluation for minimal preoperative knee flexion at initial contact and in stance is a strategy to select those cases that are not candidates for hamstring lengthening, even though the hamstrings may be tight on popliteal-angle testing. Before proceeding with hamstring lengthening, it is wise to check the calf for spasticity and the ankle for limited dorsiflexion.

## Appendices

Commentary on the physiology of early stance and late stance recurvatum

Inertia-sensitive avoidance of center of mass vaulting over the support point shortens and cushions the leg approaching mid-stance. Gastrocnemius hyper-reflexive reaction to stretch over the femoral condyle in knee extension peaks ankle resistance too soon, extending the knee via foot leverage rather than allowing knee flexion. Stiff-ankle mechanical versions of mid-stance knee extension (called ground reaction) include ankle-foot orthosis with shoe too square and toe plate too long.

Knee flexion graphs a small stance and a large swing curve. The smaller inertia-power-driven stance curve zeros out sooner with excess ground reaction and even distorts the large (yet supple) swing-phase curve. Suspect ground reaction when seeing oddities where these two curves meet.

At mid-stance, calf resistance makes the tibia/ankle/foot complex a solid unit. Three points comprised of knee center, heel tip, and metatarsal-head axis determine the leverage of ground reaction causing failure to roll.

Forward momentum raises the heel as the mass center passes midfoot. The solid triangular unit, configured for easy tipping, rolls on the forward metatarsal heads.

Beware of sensory defects which favor stability over efficiency. The trailing heel remains planted during side-to-side weight transfer. Visually, the step looks too long. The overextended trailing knee is experiencing tension (rather than compression), which is not as damaging as weight acceptance recurvatum.

Seeing sensory loss? A circle the diameter of stature will have its center at the center of mass. Both feet follow this contour during left-right weight transfer, with only two points in floor contact: leading heel and trailing forefoot. This “wheel” is seen reliably in sensation-modulated gait, but is absent in insensitive mechanisms. The wheel axle will be seen running ahead of the center of mass in robust gait-seeking speed.
